# Risk factors and predictors for tumor site origin in metastatic adenocarcinoma of unknown primary site

**DOI:** 10.1002/cam4.3684

**Published:** 2021-01-06

**Authors:** Xinrong Li, Yan Shao, Liqiang Sheng, Junquan Zhu, Zeming Wang, Kaibo Guo, Leitao Sun

**Affiliations:** ^1^ Department of Integrative Medicine & Medical Oncology Shengzhou People's Hospital (The First Affiliated Hospital of Zhejiang University Shengzhou Branch) Shengzhou Zhejiang P.R. China; ^2^ Department of Pharmacy Shengzhou People’s Hospital (The First Affiliated Hospital of Zhejiang University Shengzhou Branch) Shengzhou Zhejiang P.R. China; ^3^ The First Clinical College of Zhejiang Chinese Medical University Hangzhou Zhejiang P.R. China; ^4^ Department of Medical Oncology The First Affiliated Hospital of Zhejiang Chinese Medical University Hangzhou Zhejiang P.R. China

**Keywords:** cancer of unknown primary site, metastatic adenocarcinoma, nomogram, predictors, SEER

## Abstract

**Background:**

Metastatic adenocarcinoma of unknown primary site (MACUP) is the most common cancer of unknown primary site, and shows worse prognosis. Prediction of its tumor site origin attracts a growing attention. However, the site determined by gene expression profiling does not have a significant impact on the survival. Some other special method might need to be found out.

**Methods:**

We reviewed 1011 MACUP patients diagnosed by pathological examination and immunohistochemistry based on the Surveillance, Epidemiology, and End Results (SEER) database during 2010–2016. Kaplan–Meier curves and Cox proportional hazard model were analyzed to compare the survival. Logistic regression models and relevant nomograms were performed to predicting the probability of the primary site which including digestive system, respiratory system, and female breast. The validation and clinical utility of models were measured with relevant statistical approaches.

**Results:**

About 324 (32.1%), 299 (29.6%), and 203 (20.1%) of MACUP patients were identified as the primary sites of digestive system, respiratory system, and female breast, respectively. Patients derived from digestive system and respiratory system showed poorer survival than these with other sites. Digestive system was significantly associated with liver (Odds ratio =13.21 [95% confidence interval =8.48–21.02]) or lung (2.36 [1.40–3.97]) metastasis, while respiratory system was linked to brain (11.68 [6.68–21.26]) or lymph node (3.39 [2.26–5.13]) metastasis. Patients identified as female breast were prone to occur bone metastasis (5.85 [3.68–9.45]). Logistic regression nomograms were developed to help clinicians intuitively predict the probabilities of tumor site origin with 0.867, 0.824, and 0.753 of the C‐index, respectively. Decision curve analysis and clinical impact curves both revealed the clinical effectiveness.

**Conclusions:**

We profiled different tumor site origin of MACUP patients and established prediction models. These features might be significant for clinicians to improve the probabilities of predicting the primary sites, and to decide subsequent treatment strategy.

## Graphical abstract

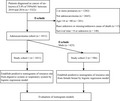

## 1 INTRODUCTION

Cancer of unknown primary site (CUP) represents a heterogeneous group with metastatic disease, for which the origin site cannot be detected despite a standardized diagnostic approach with careful examinations.^1^ It is estimated that CUP accounts for approximately 3%–5% of all newly diagnosed carcinomas^2^ and there are about 4–19 cases per 100,000 persons every year,^3^ although the exact incidence rate is hard to determine for various objective reasons. About 70%–80% of CUP histopathology is metastatic adenocarcinoma^2^, ^4^ and more than 60% patients present with metastasis in internal organs,^4^, ^5^ so metastatic adenocarcinoma of unknown primary site (MACUP) takes up the vast majority of the CUP. Patients with CUP usually receive empirical chemotherapy with a platinum‐taxane regimen,^6^ but remain poor prognosis with the median survival of approximately 6–9 months.^7^, ^8^ With the development of diagnostic method, it is more popular to formulate individualized treatment plan by gene expression profiling. However, this site‐specific therapy does not show significant difference to acquire clinical benefits.^9^ In some CUP cases that T staging is classified as T0, the primary site could be determined by pathological examination and immunohistochemistry, although no tumor site of origin could be detected by imaging examination. Compared with other CUP, the overall survival of these T0NXM1 patients is significantly prolonged.^10^

Therefore, we aimed to analyze the relationship between clinical characteristics of MACUP and different origin tumor sites determined by pathological examination and immunohistochemistry, and to study the survivals of the origin sites, based on the Surveillance, Epidemiology, and End Results (SEER) database. In this study, we focused on the primary tumor sites of digestive system, respiratory system, and female breast. First, we exhibited the differences of clinical features between these tumor sites, and calculated the survivals time. Next, some factors were found to be significantly related to the probability of the origin sites by binary logistic regression models. Finally, we intuitively predicted the primary site probabilities for MACUP patients and stratified the cases with different probabilities by constructing nomograms.

## 2 METHODS

### 2.1 Population selection and characteristics

**FIGURE 1:**
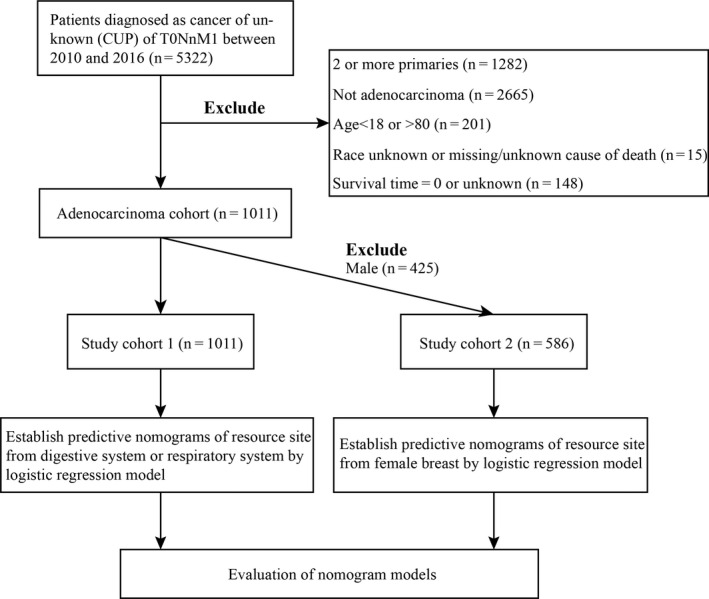
The flowchart of cases selection

The data used in the present study were extracted from the SEER 18 registry database, which involves cancer incidence and survival data and covers approximately 34.6% of the population in USA. Cases of metastatic adenocarcinoma of unknown primary site (MACUP) were identified American Joint Committee on Cancer (AJCC) stage (7th edition) “T0NxM1” and “International Classification of Diseases for Oncology, 3rd Edition (ICD‐O‐3) Hist/behav, malignant.” In addition, patients aged 18–79 years and initially diagnosed between January 2010 and December 2016, were included in the current study. In these patients, tissue or organ sources determined by pathological examination of the metastatic cancer, were recorded in “Site recode ICD‐O‐3/WHO 2008.” Cases that were missing significant information, including unknown race, unknown cause of death or unknown survival time, were subsequently excluded. In order to analyze the different sources of primary tumor site, we then divided the included MACUP patients into two groups: the study cohort 1 (whole population) for digestive system or respiratory system, and the study cohort 2 (female population) for female breast. Subsequently, the study cohorts were split into the training set and the validation set for data analysis. The flowchart of patients screening was exhibited in Figure 1. Events per variable (EPV)^11^ of prostate cancer in male population is less than ten, so we did not establish the predictive nomogram of resource site from male prostate by logistic regression model. Because the data analyzed were downloaded from the SEER database, which publicly provide open‐access and anonymized data for everyone in the world, ethical approval was unnecessary to seek for this study.

### 2.2 Outcome and variable declaration

Overall survival (OS) refers to the time from cancer diagnosis to death. Kaplan–Meier curves were performed to study the impact of the specific primary site or treatment (radiation therapy and chemotherapy). For demographic features, we included age (18–49 years, 50–64 years, 65–79 years), gender (female, male), race (white, black, other), and marital status (married, other). Tumor characteristics involved source site (digestive system, respiratory system, female breast; male prostate, gynecology system, other), lymph node metastasis (N0, Nn, Nx), liver metastasis (No, Yes), lung metastasis (No, Yes), bone metastasis (No, Yes), brain metastasis (No, Yes). Treatment‐related covariates included radiation therapy (No, Yes), chemotherapy (No, Yes), and surgery (No, Yes). Other variables involved length of follow‐up and status (alive, dead).

### 2.3 The binary logistic regression and Cox proportional hazard modeling

The logistic regression models were conducted to evaluate the probability of primary site derived from digestive system, respiratory system, or female breast, based on the training set from the study cohort 1 and study cohort 2, respectively. Candidate risk factors involved age, gender, race, marital status, tumor size, lymph node metastasis, liver metastasis, lung metastasis, lung metastasis, bone metastasis and brain metastasis. Multivariable logistic regression models were established to determine the significant risk factors. Additionally, univariable and multivariable Cox proportional hazard model were developed to study the prognostic factors of MACUP patients. Especially, in order to explore the efficacy of treatment regions on survival of patients stratified with different tumor sources, subgroup analysis displayed with forest plots was conducted by univariable Cox regression, and then, multivariable regression if the variable was significant, acquiring the HRs and 95%CIs that have corrected the bias caused by some factors.

### 2.4 Logistic regression nomograms development and validation

In order to help the physicians to predict the primary site of the whole and female MACUP patients, we constructed nomograms based on the multivariate logistic regression model of the training set. Then, patients with low‐, medium‐, and high‐risk were determined, respectively, by calculating the quantiles of scores and the difference of the specific primary site probabilities (digestive system, respiratory system, and female breast) among these subgroups was compared. Next, we calculated the concordance index (C‐index) and conducted calibration plots to perform internal validation of nomograms.^12^ The C‐index was used to evaluate the discriminatory power of the nomograms and the calibration curves were used to quantify the accuracy of the models. External validation was exhibited by calibration curves using the validation set. Moreover, we performed decision curve analysis (DCA)^13^ to reveal the clinical utility of the nomograms by calculating the net benefits at each threshold probability, while clinical impact curves (CIC)^14^ were conducted to help us understand the models’ clinical value more intuitively.

### 2.5 Statistical analysis

All data in this study were analyzed in R software (version 3.6.1, https://www.r‐proje ct.org/). The logistic regression model, nomogram, C‐index, calibration curves, DCA, CIC, Cox regression model, Kaplan–Meier curves, and forest plots were performed by using R software with packages, such as stat, rms, rmda, survival, and forestplot. *p* < 0.05 was considered statistically significant.

## 3 RESULTS

### 3.1 Population enrollment and features

We included a total of 1011 patients diagnosed with MACUP in this study, involving 425 (42%) of male and 586 (58%) of female. We found that digestive system, respiratory system, female breast, male prostate, gynecology system accounted for approximately 95% of all tumor sources determined by pathological examination of the metastatic cancer, while tumor source from breast was the most common site in female patients (Table S1). The characteristics of whole and female MACUP patients were exhibited in Table 1 and Table 2, respectively. Specifically, MACUP patients derived from digestive system were more likely to be without lymph node metastasis (60.49%) and to occur liver metastasis (63.89%), while these from respiratory system were prone to being with lymph node metastasis (59.20%) and to occur brain metastasis (35.79%). Moreover, patients with white in race (85.71%) or bone metastasis (59.61%) were significantly associated with the source site of female breast.

**TABLE 1:** Clinicopathological variables of MACUP patients derived from digestive system or respiratory system

Risk factors	Overall, *n* (%)	Digestive system, *n* (%)	Other, *n* (%)	*p*‐value	Respiratory system, *n* (%)	Other, *n* (%)	*p*‐value
	(1011)	(324)	(687)		(299)	(712)	
Age at initial diagnosis, years				0.21			0.11
18–49	106 (10.48)	42 (12.96)	64 (9.32)		22 (7.36)	84 (11.8)	
50–64	406 (40.16)	127 (39.20)	279 (40.61)		124 (41.47)	282 (39.61)	
65–79	499 (49.36)	155 (47.84)	344 (50.07)		153 (51.17)	346 (48.6)	
Gender				<0.001			<0.001
Female	586 (57.96)	157 (48.46)	429 (62.45)		137 (45.82)	449 (63.06)	
Male	425 (42.04)	167 (51.54)	258 (37.55)		162 (54.18)	263 (36.94)	
Race				0.245			0.202
White	802 (79.33)	247 (76.23)	555 (80.79)		239 (79.93)	563 (79.07)	
Black	121 (11.97)	44 (13.58)	77 (11.21)		29 (9.7)	92 (12.92)	
Other	88 (8.70)	33 (10.19)	55 (8.01)		31 (10.37)	57 (8.01)	
Marital status				0.6			0.453
Married	548 (54.20)	180 (55.56)	368 (53.57)		168 (56.19)	380 (53.37)	
Unmarried	463 (45.80)	144 (44.44)	319 (46.43)		131 (43.81)	332 (46.63)	
Node metastasis				<0.001			<0.001
N0	509 (50.35)	196 (60.49)	313 (45.56)		107 (35.79)	402 (56.46)	
Nn	374 (36.99)	69 (21.30)	305 (44.40)		177 (59.20)	197 (27.67)	
Nx	128 (12.66)	59 (18.21)	69 (10.04)		15 (5.02)	113 (15.87)	
Liver metastasis				<0.001			<0.001
No	729 (72.11)	117 (36.11)	612 (89.08)		271 (90.64)	458 (64.33)	
Yes	282 (27.89)	207 (63.89)	75 (10.92)		28 (9.36)	254 (35.67)	
Lung metastasis				<0.001			<0.001
No	847 (83.78)	244 (75.31)	603 (87.77)		279 (93.31)	568 (79.78)	
Yes	164 (16.22)	80 (24.69)	84 (12.23)		20 (6.69)	144 (20.22)	
Bone metastasis				<0.001			0.387
No	668 (66.07)	274 (84.57)	394 (57.35)		204 (68.23)	464 (65.17)	
Yes	343 (33.93)	50 (15.43)	293 (42.65)		95 (31.77)	248 (34.83)	
Brain metastasis				<0.001			<0.001
No	871 (86.15)	317 (97.84)	554 (80.64)		192 (64.21)	679 (95.37)	
Yes	140 (13.85)	7 (2.16)	133 (19.36)		107 (35.79)	33 (4.63)	
Radiation therapy				<0.001			<0.001
No	720 (71.22)	291 (89.81)	429 (62.45)		148 (49.5)	572 (80.34)	
Yes	291 (28.78)	33 (10.19)	258 (37.55)		151 (50.5)	140 (19.66)	
Chemotherapy				0.003			0.023
No	422 (41.74)	113 (34.88)	309 (44.98)		108 (36.12)	314 (44.1)	
Yes	589 (58.26)	211 (65.12)	378 (55.02)		191 (63.88)	398 (55.9)	
Surgery							<0.001
No	956 (94.56)	316 (97.53)	640 (93.16)	0.007	297 (99.33)	659 (92.56)	
Yes	55 (5.44)	8 (2.47)	47 (6.84)		2 (0.67)	53 (7.44)	
Follow‐up time	11 (4–24)	6 (2–14)	14 (5–29)	<0.001	9 (3–19)	13 (4–25)	0.002
Status				<0.001			0.005
Alive	294 (29.08)	43 (13.27)	251 (36.54)		68 (22.74)	226 (31.74)	
Dead	717 (70.92)	281 (86.73)	436 (63.46)		231 (77.26)	486 (68.26)	

Abbreviations: MACUP, Metastatic adenocarcinoma of unknown primary site.

**TABLE 2:** Clinicopathological variables of MACUP patients derived from female breast

Risk factors	Overall, *n* (%)	Female breast, *n* (%)	Other, *n* (%)	*p*‐value
	(586)	(203)	(383)	
Age at initial diagnosis, years				0.84
18–49	65 (11.09)	24 (11.82)	41 (10.70)	
50–64	232 (39.59)	82 (40.39)	150 (39.16)	
65–79	289 (49.32)	97 (47.78)	192 (50.13)	
Race				0.029
White	467 (79.69)	174 (85.71)	293 (76.50)	
Black	70 (11.95)	18 (8.87)	52 (13.58)	
Other	49 (8.36)	11 (5.42)	38 (9.92)	
Marital status				0.411
Married	288 (49.15)	105 (51.72)	183 (47.78)	
Unmarried	298 (50.85)	98 (48.28)	200 (52.22)	
Node				
N0	297 (50.68)	87 (42.86)	210 (54.83)	0.021
Nn	214 (36.52)	87 (42.86)	127 (33.16)	
Nx	75 (12.80)	29 (14.29)	46 (12.01)	
Liver metastasis				<0.001
No	429 (73.21)	169 (83.25)	260 (67.89)	
Yes	257 (26.79)	34 (16.75)	123 (32.11)	
Lung metastasis				0.675
No	500 (85.32)	171 (84.24)	329 (85.90)	
Yes	86 (14.68)	32 (15.76)	54 (14.10)	
Bone metastasis				<0.001
No	399 (68.09)	82 (40.39)	317 (82.77)	
Yes	187 (31.91)	121 (59.61)	66 (17.23)	
Brain metastasis				0.036
No	509 (86.86)	185 (91.13)	324 (84.60)	
Yes	77 (13.14)	18 (8.87)	59 (15.40)	
Radiation				
No	425 (72.53)	139 (68.47)	286 (74.67)	0.133
Yes	161 (27.47)	64 (31.53)	97 (25.33)	
Chemotherapy				0.002
No	245 (41.81)	103 (50.74)	142 (37.08)	
Yes	341 (58.19)	100 (49.26)	241 (62.92)	
Surgery				0.008
No	537 (91.64)	195 (96.06)	342 (89.30)	
Yes	49 (8.36)	8 (3.94)	41 (10.70)	
Follow‐up time, months	14 (5–29)	20 (8–37)	10 (3–23)	<0.001
Status				<0.001
Alive	201 (34.30)	94 (46.31)	107 (27.94)	
Dead	385 (65.70)	109 (53.69)	276 (72.06)	

Abbreviations: MACUP, Metastatic adenocarcinoma of unknown primary site.

### 3.2 Survival analysis and prognostic factors

**FIGURE 2:**
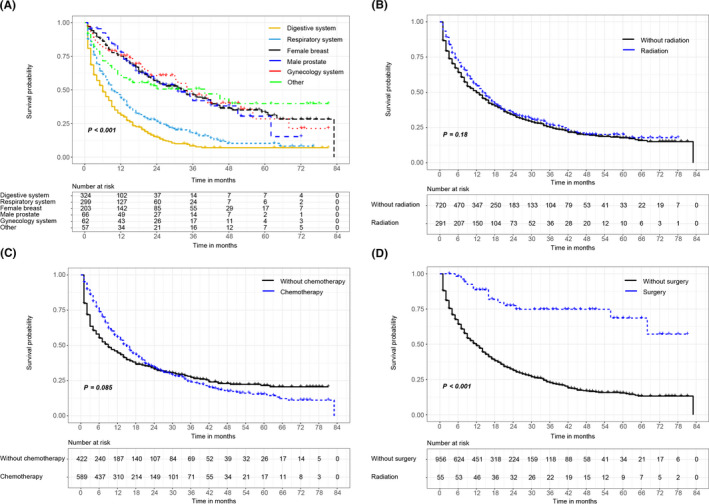
(A) Overall survival (OS) of patients with metastatic adenocarcinoma of unknown primary site (MACUP) for different tumor origin sites based on Kaplan–Meier curves; (B) OS of MACUP patients with radiation therapy or not; (C) OS of MACUP patients with chemotherapy or not; (D) OS of MACUP patients with surgery or not

MACUP patients whose primary site was at digestive system or respiratory system showed the worse prognosis, with the median OS of only 6 and 9 months, respectively, when compared to the source sites of female breast (33 months), male prostate (31 months), gynecology system (34 months), and other (40 months) (Figure 2A). Kaplan–Meier curves also revealed that radiation therapy and chemotherapy did not affect the OS, while surgery might prolong the OS, although only 55 patients underwent the resection (Figure 2B–D).

For the results of the Cox regression, we found that patients with older age (hazard ratio (HR) = 1.68, 95% confidence interval (95%CI) = 1.29–2.20, *p* < 0.001), male (HR = 1.22, 95%CI = 1.02–1.44, *p* = 0.025), lymph node metastasis (HR = 1.22, 95%CI = 1.03–1.46, *p* = 0.021) or unknown status (HR = 1.42, 95%CI = 1.14–1.77, *p* = 0.002), and liver metastasis (HR = 1.40, 95%CI = 1.15–1.70, *p* < 0.001) experienced poorer prognosis, while patients with the source site of female breast (HR = 0.37, 95%CI = 0.29–0.49, *p* < 0.001) and male prostate (HR = 0.32, 95%CI = 0.22–0.47, *p* < 0.001), gynecology system (HR = 0.51, 95%CI = 0.34–0.76, *p* = 0.001), as well as those with surgery (HR = 0.27, 95%CI = 0.17–0.47, *p* < 0.001) showed favorable survival (Table 3).

**TABLE 3:** Cox regression analysis of the prognostic factors for MACUP patients

Risk factors	Univariate Cox regression		Multivariate Cox regression	
	HR* (95% CI*)	*p*‐value	HR* (95% CI*)	*p*‐value
Age at initial diagnosis, years				0.229
18–49	Reference		Reference	
50–64	1.29 (0.99–1.69)	0.061	1.30 (1.00–1.71)	0.054
65–79	1.56 (1.20–2.03)	<0.001	1.68 (1.29–2.20)	<0.001
Gender				
Female	Reference		Reference	
Male	1.58 (1.37–1.84)	<0.001	1.22 (1.02–1.44)	0.025
Race				
White	Reference			
Black	1.06 (0.84–1.33)	0.637		
Other	1.01 (0.78–1.31	0.935		
Marital status				
Married	Reference			
Unmarried	1.07 (0.92–1.24)	0.37		
Source site				
Digestive system	Reference		Reference	
Respiratory system	0.71 (0.60–0.85)	<0.001	0.80 (0.64–1.00)	0.052
Female breast	0.30 (0.24–0.38)	<0.001	0.37 (0.29–0.49)	<0.001
Male prostate	0.30 (0.21–0.43)	<0.001	0.32 (0.22–0.47)	<0.001
Gynecology system	0.30 (0.21–0.43)	<0.001	0.51 (0.34–0.76)	0.001
Other	0.32 (0.22–0.47)	<0.001	0.54 (0.36–0.80)	0.002
Node metastasis				
N0	Reference		Reference	
Nn	1.13 (0.96–1.33)	0.132	1.22 (1.03–1.46)	0.021
Nx	1.57 (1.26–1.94)	<0.001	1.42 (1.14–1.77)	0.002
Liver metastasis				
No	Reference		Reference	
Yes	2.03 (1.74–2.38)	<0.001	1.40 (1.15–1.70)	<0.001
Lung metastasis				
No	Reference		Reference	
Yes	1.32 (1.09–1.60)	0.004	1.11 (0.91–1.35)	0.319
Bone metastasis				
No	Reference			
Yes	0.88 (0.75–1.03)	0.105		
Brain metastasis				
No	Reference			
Yes	1.19 (0.97–1.46)	0.104		
Radiation				
No	Reference			
Yes	0.89 (0.76–1.05)	0.165		
Chemotherapy				
No	Reference			
Yes	0.87 (0.75–1.01)	0.0621		
Surgery				
No	Reference		Reference	
Yes	0.20 (0.12–0.34)	<0.001	0.27 (0.17–0.47)	<0.001

Abbreviations: MACUP, Metastatic adenocarcinoma of unknown primary site.

**FIGURE 3:**
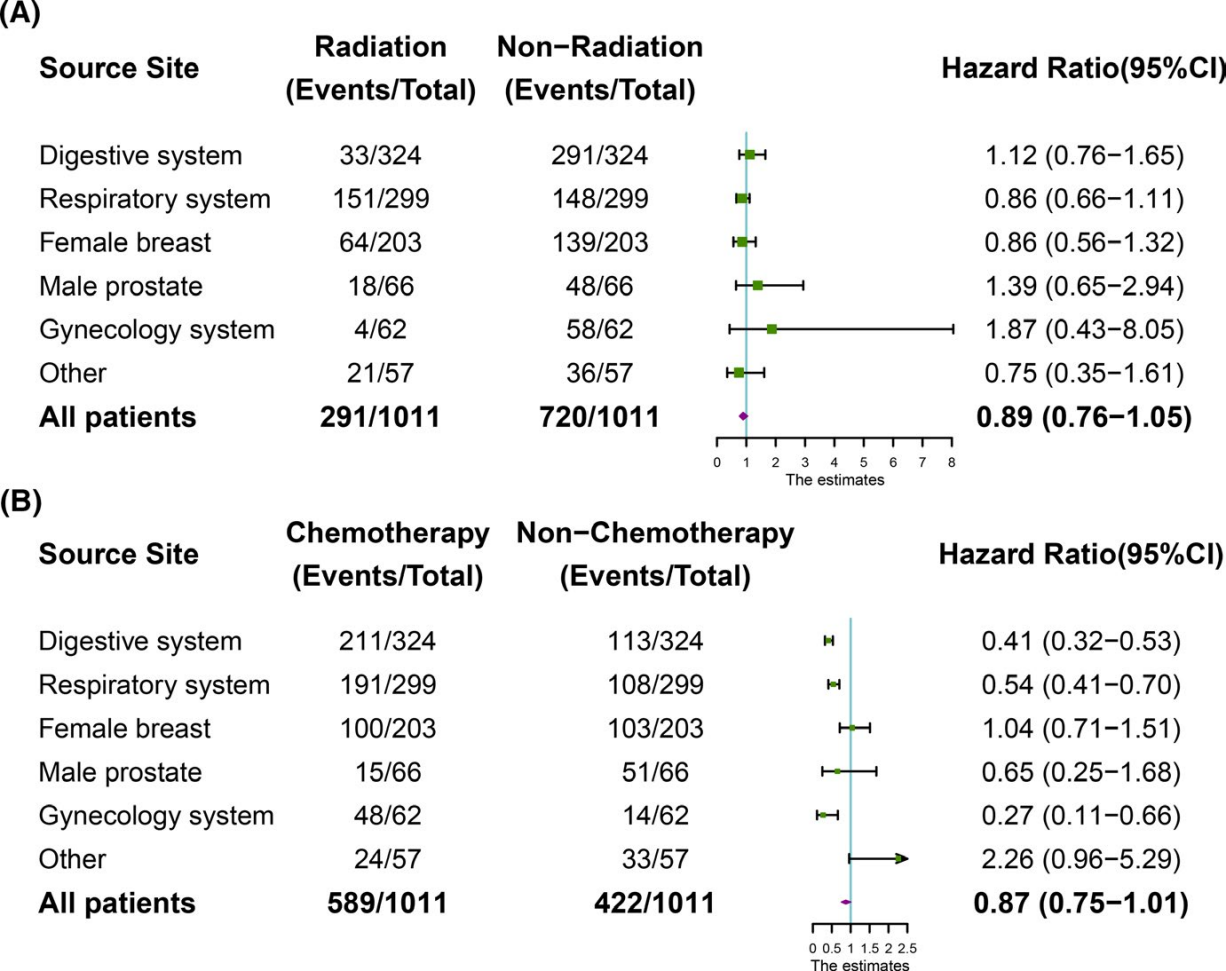
(A) Harzad Ratios (HRs) of radiation therapy for different tumor origin sites based on forest plots; (B) Harzad Ratios (HRs) of chemotherapy for different tumor origin sites

Considering the sample size, we conducted the subgroup analysis stratified by the primary tumor sites to determine the impact of radiation therapy or chemotherapy on the prognosis. Similarly, there was no significant difference for patients with different sites between radiation therapy group and nonradiation group (Figure 3A). Interestingly, patients derived from digestive system (HR = 0.41, 95%CI = 0.32–0.53, *p* < 0.001), respiratory system (HR = 0.54, 95%CI = 0.41–0.70, *p* < 0.001), or gynecology system (HR = 0.27, 95%CI = 0.11–0.66, *p* < 0.001) could benefit from the chemotherapy (Figure 3B).

### 3.3 Risk factors of the specific source sites and prediction models

The study cohorts were further divided into the training and the validation sets, the characteristics of which were exhibited in Table S2 and S3. Risk predictors for the source sites of digestive system, respiratory system, or female breast were estimated by using binary logistic regression models, and the results of the characteristics were provided in Tables 3, 4, 5, respectively. Male (OR = 2.24, 95%CI = 1.48–3.44, *p* < 0.001), liver metastasis (OR = 13.21, 95%CI = 8.48–21.02, *p* < 0.001), lung metastasis (OR = 2.36, 95%CI = 1.40–3.97, *p* = 0.001), lymph node metastasis (OR = 0.43, 95%CI = 0.27–0.69, *p* < 0.001), bone metastasis (OR = 0.21, 95%CI = 0.130.34, *p* < 0.001), and brain metastasis (OR = 0.09, 95%CI = 0.03–0.22, *p* < 0.001) were significantly related to the source site of digestive system (Table 4), while older age (50–64 years: OR = 2.29, 95%CI = 1.10–5.08, *p* = 0.033; 65–79 years: OR = 2.32, 95%CI = 1.12–5.10, *p* = 0.028), male (OR = 2.14, 95%CI = 1.45–3.20, *p* < 0.001), lymph node metastasis (OR = 3.39, 95%CI = 2.26–5.13, *p* < 0.001), brain metastasis (OR = 11.68, 95%CI = 6.68–21.26, *p* < 0.001), liver metastasis (OR = 0.24, 95%CI = 0.13–0.41, *p* < 0.001), and lung metastasis (OR = 0.29, 95%CI = 0.15–0.53, *p* < 0.001) were significantly linked to the primary tumor site of respiratory system (Table 5). Female MACUP patients with black race (OR = 0.37, 95%CI = 0.24–0.54, *p* = 0.016), unknown status of lymph node metastasis (OR = 2.94, 95%CI = 1.43–6.09, *p* = 0.003), bone metastasis (OR = 5.85, 95%CI = 3.68–9.45, *p* < 0.001), and liver metastasis (OR = 0.36, 95%CI = 0.20–0.62, *p* < 0.001) were associated with the site of breast (Table 6).

**TABLE 4:** Logistic regression analysis of the risk factors for the source site of digestive system in MACUP patients

Risk factors	Univariate logistic regression		Multivariate logistic regression	
	OR* (95% CI*)	*p*‐value	OR* (95% CI*)	*p*‐value
Age at initial diagnosis, years				
18–49	Reference			
50–64	0.73 (0.42–1.26)	0.245		
65–79	0.70 (0.41–1.20)	0.189		
Gender				
Female	Reference		Reference	
Male	1.93 (1.40–2.66)	<0.001	2.24 (1.48–3.44)	<0.001
Race				
White	Reference			
Black	1.24 (0.78–1.94)	0.36		
Other	1.39 (0.79–2.38)	0.242		
Marital status				
Married	Reference			
Unmarried	0.90 (0.65–1.24)	0.522		
Node metastasis				
N0	Reference		Reference	
Nn	0.48 (0.33–0.70)	<0.001	0.43 (0.27–0.69)	<0.001
Nx	1.45 (0.91–2.30)	0.118	0.75 (0.40–1.39)	0.367
Liver metastasis				
No	Reference		Reference	
Yes	14.59 (9.88–21.87)	<0.001	13.21 (8.48–21.02)	<0.001
Lung metastasis				
No	Reference		Reference	
Yes	2.17 (1.45–3.25)	<0.001	2.36 (1.40–3.97)	0.001
Bone metastasis				
No	Reference		Reference	
Yes	0.29 (0.20–0.43)	<0.001	0.21 (0.13–0.34)	<0.001
Brain metastasis				
No	Reference		Reference	
Yes	0.10 (0.03–0.22)	<0.001	0.09 (0.03–0.22)	<0.001

Abbreviations: MACUP, Metastatic adenocarcinoma of unknown primary site.

**TABLE 5:** Logistic regression analysis of the risk factors for the source site of respiratory system in MACUP patients

Risk factors	Univariate logistic regression		Multivariate logistic regression	
	OR* (95% CI*)	*p*‐value	OR* (95% CI*)	*p*‐value
Age at initial diagnosis, years				
18–49	Reference		Reference	
50–64	1.91 (1.02–3.79)	0.052	2.29 (1.10–5.08)	0.033
65–79	1.98 (1.07–3.92)	0.038	2.32 (1.12–5.10)	0.028
Gender				
Female	Reference		Reference	
Male	1.57 (1.13–2.17)	0.007	2.14 (1.45–3.20)	<0.001
Race				
White	Reference			
Black	0.77 (0.46–1.25)	0.309		
Other	1.23 (0.70–2.14)	0.46		
Marital status				
Married	Reference			
Unmarried	0.89 (0.64–1.23)	0.486		
Node metastasis				
N0	Reference		Reference	
Nn	2.87 (2.03–4.07)	<0.001	3.39 (2.26–5.13)	<0.001
Nx	0.41 (0.19–0.80)	0.013	0.59 (0.26–1.22)	0.177
Liver metastasis				
No	Reference		Reference	
Yes	0.19 (0.11–0.30)	<0.001	0.24 (0.13–0.41)	<0.001
Lung metastasis				
No	Reference		Reference	
Yes	0.30 (0.16–0.51)	<0.001	0.29 (0.15–0.53)	<0.001
Bone metastasis				
No	Reference			
Yes	0.74 (0.52–1.05)	0.096		
Brain metastasis				
No	Reference		Reference	
Yes	10.37 (6.38–17.39)	<0.001	11.68 (6.68–21.26)	<0.001

Abbreviations: MACUP, Metastatic adenocarcinoma of unknown primary site.

**TABLE 6:** Logistic regression analysis of the risk factors for the source site of female breast in MACUP patients

Risk factors	Univariate logistic regression		Multivariate logistic regression	
	OR* (95% CI*)	*p*‐value	OR* (95% CI*)	*p*‐value
Age at initial diagnosis, years				
18–49	Reference			
50–64	0.93 (0.48–1.83)	0.839		
65–79	0.83 (0.43–1.61)	0.572		
Race				
White	Reference		Reference	
Black	0.46 (0.22–0.91)	0.033	0.37 (0.24–0.54)	0.016
Other	0.64 (0.37–1.76)	0.637	0.59 (0.24–1.37)	0.224
Marital status				
Married	Reference			
Unmarried	0.94 (0.63–1.41)	0.78		
Node metastasis				
N0	Reference		Reference	
Nn	1.62 (1.05–2.50)	0.03	1.32 (0.82–2.15)	0.256
Nx	1.86 (1.00–3.46)	0.05	2.94 (1.43–6.09)	0.003
Liver metastasis				
No	Reference		Reference	
Yes	0.39 (0.24–0.63)	<0.001	0.36 (0.20–0.62)	<0.001
Lung metastasis				
No	Reference			
Yes	1.09 (0.63–1.87)	0.746		
Bone metastasis				
No	Reference		Reference	
Yes	5.72 (3.68–9.00)	<0.001	5.85 (3.68–9.45)	<0.001
Brain metastasis				
No	Reference			
Yes	0.59 (0.30–1.11)	0.112		

Abbreviations: MACUP, Metastatic adenocarcinoma of unknown primary site.

**FIGURE 4:**
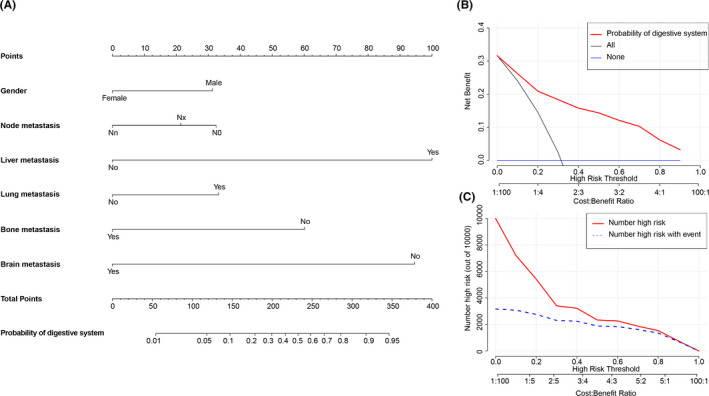
(A) Logistic‐regression nomogram for predicting the site probability of digestive system in patients with metastatic adenocarcinoma of unknown primary site (MACUP). There are six factors in this nomogram, involving gender, lymph node metastasis, liver metastasis, lung metastasis, bone metastasis and brain metastasis; (B) Decision curve analysis shows that if the threshold probability was between 1% and 90%, then using the nomogram to predict the site probability of digestive system in MACUP patients added more clinical benefits; (C) Clinical impact curve reveals that the number of high risk increases as the threshold probability increases, indicating that the nomogram can provide good clinical utility in MACUP patients

**FIGURE 5:**
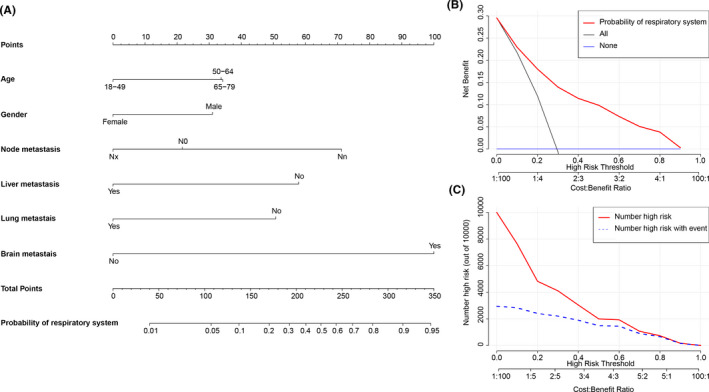
(A) Logistic‐regression nomogram for predicting the site probability of respiratory system in patients with metastatic adenocarcinoma of unknown primary site (MACUP). There are six factors in this nomogram, involving age, gender, lymph node metastasis, liver metastasis, lung metastasis and brain metastasis; (B) Decision curve analysis shows that if the threshold probability was between 1% and 90%, then using the nomogram to predict the site probability of respiratory system in MACUP patients added more clinical benefits; (C) Clinical impact curve reveals that the number of high risk increases as the threshold probability increases, indicating that the nomogram can provide good clinical utility in MACUP patients

**FIGURE 6:**
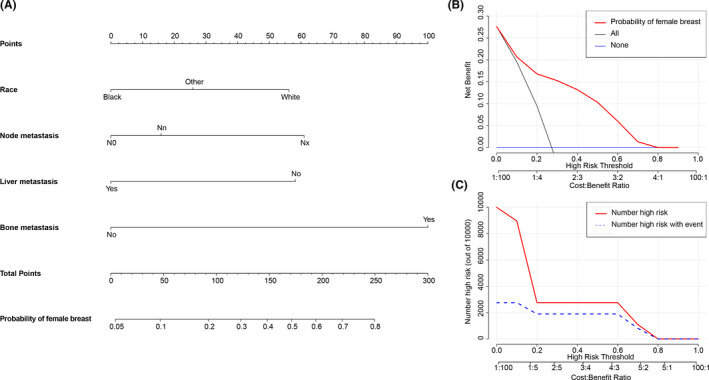
(A) Logistic‐regression nomogram for predicting the site probability of female breast in patients with metastatic adenocarcinoma of unknown primary site (MACUP). There are four factors in this nomogram, involving race, lymph node metastasis, liver metastasis and bone metastasis; (B) Decision curve analysis shows that if the threshold probability was between 1% and 80%, then using the nomogram to predict the site probability of female breast in MACUP patients added more clinical benefits; (C) Clinical impact curve reveals that the number of high risk increases as the threshold probability increases, indicating that the nomogram can provide good clinical utility in MACUP patients

**FIGURE 7:**
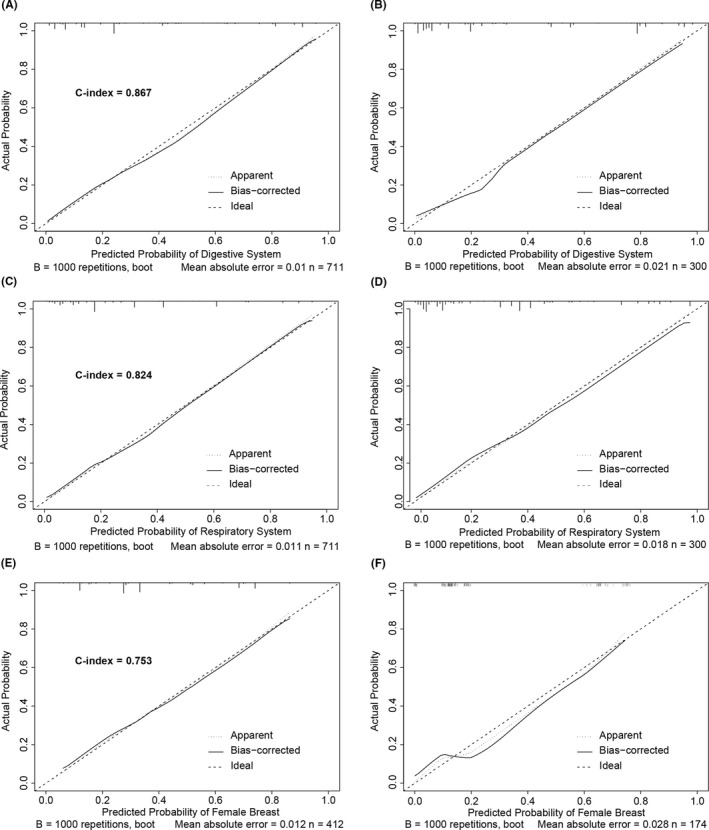
(A) Calibration curve and C‐index of 0.867 show good accuracy and discrimination based on the internal validation for the prediction of digestive system; (B) Calibration curve shows good accuracy based on the external validation for the prediction of digestive system; (C) Calibration curve and C‐index of 0.824 shows good accuracy and discrimination based on the internal validation for the prediction of respiratory system; (D) Calibration curve shows good accuracy based on the external validation for the prediction of respiratory system; (E) Calibration curve and C‐index of 0.824 shows good accuracy and discrimination based on the internal validation for the prediction of female breast; (F) Calibration curve shows good accuracy based on the external validation for the prediction of female breast

Logistic regression nomograms were constructed on the basis of the significant factors to predict for the source sites of digestive system (Figure 4A) or respiratory system (Figure 5A) in whole MACUP patients, and for the source sites of breast in female patients (Figure 6A). The C‐index of these nomograms with 86.7%, 82.4%, and 75.3%, respectively, showed good discrimination, while excellent accuracy of the models was revealed by the calibration curves, regardless of the internal or external validation (Figure 7A–F). In addition, the risk scores of some covariates were calculated and exhibited in Table S4, identifying low‐, medium‐, and high‐risk patients by using the 25th and 75th the quantiles of scores. The risk stratification indicated that the nomograms showed significant difference of site probability between these subgroups (Figure S1). Furthermore, DCAs were conducted based on the logistic‐regression nomograms and showed the proper threshold probabilities for predicting the source site of digestive system (0%–90%) (Figure 4B), respiratory system (0%–90%) (Figure 5B), or female breast (0%–80%) (Figure 6B). CICs revealed that the number of high‐risk patients evaluated by these three models would be closer to the number of high‐risk events, as threshold probabilities increased (Figures 4C, 5C, and 6C).

## 4 DISCUSSION

Cancer of unknown primary site (CUP) is considered as a mysterious malignant tumor and its prognosis is poorer than metastatic cancers with clear primary site.^15^ Pathological examination and gene expression profiling detected to determine the tissue of origin are attracting the wide attention.^16^, ^17^ Theoretically, the treatment of CUP should depend on the tumor‐site origin, and site‐specific therapy rather than a nonselective empirical chemotherapy would be better to conducted individually. However, whether precision therapy can bring survival benefits is controversial based on current evidence. Hayashi et al.^18^ and Fizazi et al.^19^ conducted randomized controlled trials and found that site‐specific therapy based on microarray profiling could not improve the survival, meanwhile it would cost a lot.

According to the definition, Tao et al. classified CUP into two states^10^: Type 1 refers that tissue of origin cannot be determined by pathological examination and immunohistochemistry of the metastatic cancer; Type 2 means that tissue of origin can be identified by pathological examination and immunohistochemistry of the metastatic cancer, resulting in that the T staging is defined as T0. Moreover, it was reported that type 2 of CUP exhibited better prognosis than the type 1 in the study of Tao et al.^10^ Metastatic adenocarcinoma of unknown primary site (MACUP) is the most common CUP^20^ and signals an unfavorable prognosis.^4^ Considering the data availability, we planned to include the MACUP patients whose potential primary sites were identified by pathological examination and immunohistochemistry, and next profiled their demographic variables and tumor characteristics. Especially, the probabilities of digestive system, respiratory system, or female breast were displayed by our proposed nomograms, respectively, which might have an impact on the clinical strategy.

1011 patients with MACUP were involved in the present study. Based on the detection of pathological examination and immunohistochemistry, digestive system (32.05%), respiratory system (29.57%), and female breast (20.08%) were the three most common sites, which was similar to the cancers identified at the definitely primary sites.^21^ As for the prognosis of different origin sites, we found that digestive system or respiratory system showed the worst survival in the Kaplan–Meier curves and multivariate Cox regression, which was also consistent with the carcinomas whose primary sites were determined at the initial diagnosis time.^21^ Interestingly, radiation and chemotherapy were not significantly associated with the prognosis, while surgery for existed tumors could prolong the survival time, indicating that MACUP was so heterogeneous that radiation or chemotherapy could not accurately treat the primary tumor. Furthermore, subgroup analysis was conducted and the result showed that it was necessary for MACUP patients derived from digestive or respiratory system to accept chemotherapy, which might be due to their composition of colon cancer and lung cancer as well as high sensitivity of the chemotherapy.

Considering the sample size, we performed three logistic regression models to identify risk factors for the origin sites of digestive system, respiratory system, and female breast. We found that metastatic features were significantly associated with different primary sites. MACUP patients derived from digestive system were more likely to be with liver or lung metastasis and without lymph node, bone, or brain metastasis, while lymph node or brain metastasis and nonliver or nonlung metastasis were more prone to be existed in respiratory system. For the origin site in female breast, lymph node, bone, or nonliver metastasis were more common. However, these results were not reported before and we first revealed the phenomena, which might provide some reference for clinical practice.

Finally, regarding the models conducted by logistic regression and variables screening, we constructed three relevant nomograms for predicting the different origin sites, including digestive system, respiratory system, and female breast. These nomograms could be effective to predict the probabilities of the primary site for MACUP patients by using common clinical features, with the high c‐index and excellent calibration. Moreover, the clinical effectiveness of our nomograms was evaluated by DCAs and CICs, revealing good utility in the large range of threshold probability, which could help clinicians identify MACUP as the potential site‐determined carcinomas according their probabilities. Additionally, subsequent site‐specific therapy would be tailored individually by referring to the protocol of the known tumor site, and these patients could benefit from this treatment model.^10^

Nevertheless, there are still some shortcomings and limitations in this study. First, it was a retrospective analysis based on the SEER database, and the current determination of the origin sites by pathological examination and immunohistochemistry lacked a specific standard, which might be due to the complexity of MACUP and the future direction of prediction for the primary sites. Second, gene testing might be important to clarify the heterogeneity of MACUP as a mysterious carcinoma. Although these common clinical features could help physicians calculate the probabilities of the specific sites, directly distinguishing the one origin site from another might be difficult and further research should be conducted. Finally, it is necessary for our models to be validated by another external populations, despite their validation in the present study showed excellent consistency.

## CONFLICT OF INTEREST

The authors declare no conflict of interest.

## Funding information

General Research Program for Education of Zhejiang Provincial (Leitao Sun, No. Y202045212).

## Supporting information

Figure S1

Table S1–S4

## Data Availability

The data sets generated for this study are available in the SEER database (https://seer.cancer.gov/about/overview.html).
