# Supplementary figures and images for: Risk factors and predictors for tumor site origin in metastatic adenocarcinoma of unknown primary site
Source: Cancer Med. 2021 Jan 6;10(3):974–88. doi: 10.1002/cam4.3684 (PMC7897950; doi:10.1002/cam4.3684)

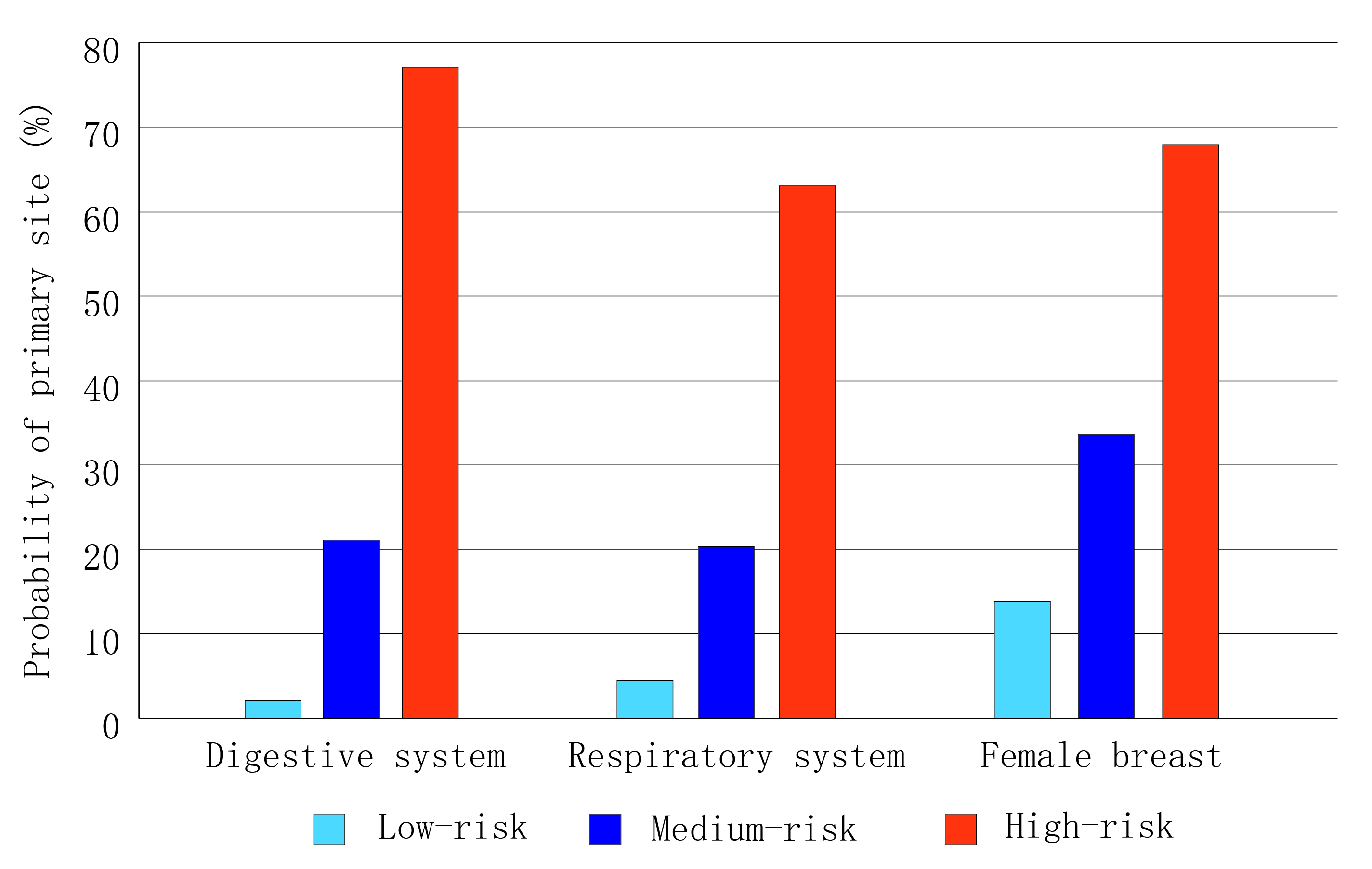

Supplement: Supplementary file 1 — Figure S1 [file CAM4-10-974-s001.tif]
