# Supplementary material for: Risk factors and predictors for tumor site origin in metastatic adenocarcinoma of unknown primary site
Source: Cancer Med. 2021 Jan 6;10(3):974–88. doi: 10.1002/cam4.3684 (PMC7897950; doi:10.1002/cam4.3684)
Supplement: Supplementary file 2 — Table S1–S4 [file CAM4-10-974-s002.docx]

**Supplement Table 1 Tumor origin sites of MACUP patients**

| **Classification** | **Site recode ICD-O-3/WHO 2008** | **Number** |
| --- | --- | --- |
| **Digestive system** | Large Intestine, NOS | 129 |
|  | Pancreas | 122 |
|  | Stomach | 20 |
|  | Gallbladder | 15 |
|  | Rectosigmoid Junction | 14 |
|  | Small Intestine | 6 |
|  | Other Biliary | 5 |
|  | Rectum | 3 |
|  | Appendix | 3 |
|  | Cecum | 2 |
|  | Sigmoid Colon | 2 |
|  | Transverse Colon | 1 |
|  | Esophagus | 1 |
|  | Ascending Colon | 1 |
| **Respiratory system** | Lung and Bronchus | 299 |
| **Female breast** | Female breast | 203 |
| **Male prostate** | Prostate | 66 |
| **Gynecology system** | Ovary | 53 |
|  | Corpus Uteri | 7 |
|  | Cervix Uteri | 1 |
|  | Uterus, NOS | 1 |
| **Other** | Kidney and Renal Pelvis | 28 |
|  | Thyroid | 17 |
|  | Salivary Gland | 4 |
|  | Male breast | 4 |
|  | Peritoneum, Omentum and Mesentery | 3 |
|  | Urinary Bladder | 1 |

Abbreviations: MACUP= Metastatic adenocarcinoma of unknown primary site.

**Supplement Table 2 Clinicopathological variables of the whole MACUP patients stratified by the training set and the validation set**

| **Risk factors** |  |  |  |  |
| --- | --- | --- | --- | --- |
|  | **Overall, n (%)** | **Training set, n (%)** | **Validation set, n (%)** | ***P*-value** |
|  | **(1011)** | **(711)** | **(300)** |  |
| **Age at initial diagnosis, years** |  |  |  | 0.229 |
| 18-49 | 106 (10.48) | 70 (9.85) | 36 (12.00) |  |
| 50–64 | 406 (40.16) | 297 (41.77) | 109 (36.33) |  |
| 65–79 | 499 (49.36) | 344 (48.38) | 155 (51.67) |  |
| **Gender** |  |  |  | 0.139 |
| Female | 586 (57.96) | 401 (56.40) | 185 (61.67) |  |
| Male | 425 (42.04) | 310 (43.60) | 115 (38.33) |  |
| **Race** |  |  |  | 0.041 |
| White | 802 (79.33) | 553 (77.78) | 249 (83.00) |  |
| Black | 121 (11.97) | 97 (13.64) | 24 (8.00) |  |
| Other | 88 (8.70) | 61 (8.58) | 27 (9.00) |  |
| **Marital status** |  |  |  | 0.398 |
| Married | 548 (54.20) | 392 (55.13) | 156 (52.00) |  |
| Unmarried | 463 (45.80) | 319 (44.87) | 144 (48.00) |  |
| **Source site** |  |  |  | 0.031 |
| Digestive system | 324 (32.05) | 225 (31.65) | 99 (33.00) |  |
| Respiratory system | 299 (29.57) | 210 (29.54) | 89 (29.67) |  |
| Female breast | 203 (20.08) | 131 (18.42) | 72 (24.00) |  |
| Male prostate | 66 (6.53) | 57 (8.02) | 9 (3.00) |  |
| Gynecology system | 62 (6.13) | 46 (6.47) | 16 (5.33) |  |
| Other | 57 (5.64) | 42 (5.91) | 15 (5.00) |  |
| **Node metastasis** |  |  |  | 0.518 |
| N0 | 509 (50.35) | 364 (51.20) | 145 (48.33) |  |
| Nn | 374 (36.99) | 255 (35.86) | 119 (39.67) |  |
| Nx | 128 (12.66) | 92 (12.94) | 36 (12.00) |  |
| **Liver metastasis** |  |  |  | 0.459 |
| No | 729 (72.11) | 518 (72.86) | 211 (70.33) |  |
| Yes | 282 (27.89) | 193 (27.14) | 89 (29.67) |  |
| **Lung metastasis** |  |  |  |  |
| No | 847 (83.78) | 593 (83.40) | 254 (84.67) | 0.686 |
| Yes | 164 (16.22) | 118 (16.60) | 46 (15.33) |  |
| **Bone metastasis** |  |  |  | 0.534 |
| No | 668 (66.07) | 465 (65.40) | 203 (67.67) |  |
| Yes | 343 (33.93) | 246 (34.60) | 97 (32.33) |  |
| **Brain metastasis** |  |  |  | 0.697 |
| No | 871 (86.15) | 615 (86.50) | 256 (85.33) |  |
| Yes | 140 (13.85) | 96 (13.50) | 44 (14.67) |  |
| **Radiation** |  |  |  | 0.178 |
| No | 720 (71.22) | 497 (69.90) | 223 (74.33) |  |
| Yes | 291 (28.78) | 214 (30.10) | 77 (25.67) |  |
| **Chemotherapy** |  |  |  | 0.02 |
| No | 422 (41.74) | 314 (44.16) | 108 (36.00) |  |
| Yes | 589 (58.26) | 397 (55.84) | 192 (64.00) |  |
| **Surgery** |  |  |  |  |
| No | 956 (94.56) | 669 (94.09) | 287 (95.67) | 0.392 |
| Yes | 55 (5.44) | 42 (5.91) | 13 (4.33) |  |
| **Follow-up time, months** | 11 (4-24) | 11 (4-24) | 11 (3-23) | 0.902 |
| **Status** |  |  |  | 0.384 |
| Alive | 294 (29.08) | 213 (29.96) | 81 (27.00) |  |
| Dead | 717 (70.92) | 498 (70.04) | 219 (73.00) |  |

Abbreviations: MACUP= Metastatic adenocarcinoma of unknown primary site.

**Supplement Table 3 Clinicopathological variables of the female MACUP patients stratified by the training set and the validation set**

| **Risk factors** |  |  |  |  |
| --- | --- | --- | --- | --- |
|  | **Overall, n (%)** | **Training cohort, n (%)** | **Validation cohort, n (%)** | ***P*-value** |
|  | **(586)** | **(412)** | **(174)** |  |
| **Age at initial diagnosis, years** |  |  |  | 0.833 |
| 18-49 | 65 (11.09) | 47 (11.41) | 18 (10.34) |  |
| 50–64 | 232 (39.59) | 165 (40.05) | 67 (38.51) |  |
| 65–79 | 289 (49.32) | 200 (48.54) | 89 (51.15) |  |
| **Race** |  |  |  | 0.401 |
| White | 467 (79.69) | 334 (81.07) | 133 (76.44) |  |
| Black | 70 (11.95) | 47 (11.41) | 23 (13.22) |  |
| Other | 49 (8.36) | 31 (7.52) | 18 (10.34) |  |
| **Marital status** |  |  |  | 0.277 |
| Married | 288 (49.15) | 209 (50.73) | 79 (45.40) |  |
| Unmarried | 298 (50.85) | 203 (49.27) | 95 (54.60) |  |
| **Source site** |  |  |  | 0.208 |
| Female breast | 203 (34.64) | 155 (37.62) | 48 (27.59) |  |
| Digestive system | 157 (26.79) | 108 (26.21) | 49 (28.16) |  |
| Respiratory system | 137 (23.38) | 91 (22.09) | 46 (26.44) |  |
| Gynecology system | 62 (10.58) | 40 (9.71) | 22 (16.64) |  |
| Other | 27 (4.61) | 18 (4.37) | 9 (5.17) |  |
| **Node metastasis** |  |  |  | 0.464 |
| N0 | 297 (50.68) | 203 (49.27) | 94 (54.02) |  |
| Nn | 214 (36.52) | 157 (38.11) | 57 (32.76) |  |
| Nx | 75 (12.80) | 52 (12.62) | 23 (13.22) |  |
| **Liver metastasis** |  |  |  | 0.212 |
| No | 429 (73.21) | 295 (71.60) | 134 (77.01) |  |
| Yes | 257 (26.79) | 117 (28.40) | 40 (22.99) |  |
| **Lung metastasis** |  |  |  |  |
| No | 500 (85.32) | 346 (83.98) | 154 (88.51) | 0.198 |
| Yes | 86 (14.68) | 66 (16.02) | 20 (11.49) |  |
| **Bone metastasis** |  |  |  | 0.435 |
| No | 399 (68.09) | 276 (66.99) | 123 (70.69) |  |
| Yes | 187 (31.91) | 136 (33.01) | 51 (29.31) |  |
| **Brain metastasis** |  |  |  | 0.48 |
| No | 509 (86.86) | 361 (87.62) | 148 (85.06) |  |
| Yes | 77 (13.14) | 51 (12.38) | 26 (14.94) |  |
| **Radiation** |  |  |  | 0.585 |
| No | 425 (72.53) | 302 (73.30) | 123 (70.69) |  |
| Yes | 161 (27.47) | 110 (26.70) | 51 (29.31) |  |
| **Chemotherapy** |  |  |  | 0.819 |
| No | 245 (41.81) | 174 (42.23) | 71 (40.80) |  |
| Yes | 341 (58.19) | 238 (57.77) | 103 (59.20) |  |
| **Surgery** |  |  |  |  |
| No | 537 (91.64) | 377 (91.50) | 160 (91.95) | 0.987 |
| Yes | 49 (8.36) | 35 (8.50) | 14 (8.05) |  |
| **Follow-up time, months** | 14 (5-29) | 14 (5-30) | 13 (4-26.75) | 0.115 |
| **Status** |  |  |  | 0.678 |
| Alive | 201 (34.30) | 144 (34.95) | 57 (32.76) |  |
| Dead | 385 (65.70) | 268 (65.05) | 117 (67.24) |  |

Abbreviations: MACUP= Metastatic adenocarcinoma of unknown primary site.

**Supplement Table 4 Nomogram score of independent factors for different source sites in MACUP patients**

| **Risk factors** |  |  |  |
| --- | --- | --- | --- |
|  |  |  |  |
|  | **Digestive system** | **Respiratory system** | **Female breast** |
| **Age at initial diagnosis, years** |  |  |  |
| 18-49 |  | 0 |  |
| 50–64 |  | 34 |  |
| 65–79 |  | 34 |  |
| **Gender** |  |  |  |
| Female | 0 | 0 |  |
| Male | 31 | 31 |  |
| **Race** |  |  |  |
| White |  |  | 56 |
| Black |  |  | 0 |
| Other |  |  | 26 |
| **Node metastasis** |  |  |  |
| N0 | 32 | 22 | 0 |
| Nn | 0 | 71 | 16 |
| Nx | 21 | 0 | 61 |
| Liver metastasis |  |  |  |
| No | 0 | 58 | 58 |
| Yes | 100 | 0 | 0 |
| Lung metastasis |  |  |  |
| No | 0 | 51 |  |
| Yes | 33 | 0 |  |
| Bone metastasis |  |  |  |
| No | 60 |  | 0 |
| Yes | 0 |  | 100 |
| Brain metastasis |  |  |  |
| No | 95 | 0 |  |
| Yes | 0 | 100 |  |

Abbreviations: MACUP= Metastatic adenocarcinoma of unknown primary site.
